# A class of carbonic anhydrase IX/XII – selective carboxylate inhibitors

**DOI:** 10.1080/14756366.2020.1715388

**Published:** 2020-01-22

**Authors:** Rakia Abd Alhameed, Emanuela Berrino, Zainab Almarhoon, Ayman El-Faham, Claudiu T. Supuran

**Affiliations:** a Department of Chemistry, College of Science, King Saud University, Riyadh, Saudi Arabia; b Dipartimento Neurofarba, Sezione di Scienze Farmaceutiche e Nutraceutiche, Università degli Studi di Firenze, Florence, Italy; c Chemistry Department, Faculty of Science, Alexandria University, Alexandria, Egypt

**Keywords:** Carbonic anhydrase, inhibitor, isoform XII, isoform-selective inhibitor, carboxylate

## Abstract

A small series of 2,4-dioxothiazolidinyl acetic acids was prepared from thiourea, chloroacetic acid, aromatic aldehydes, and ethyl-2-bromoacetate. They were assayed for the inhibition of four physiologically relevant carbonic anhydrase (CA, EC 4.2.1.1) isoforms of human (h) origin, the cytosolic hCA I and II, and the transmembrane hCA IX and XII, involved among others in tumorigenesis (hCA IX and XII) and glaucoma (hCA II and XII). The two cytosolic isoforms were not inhibited by these carboxylates, which were also rather ineffective as hCA IX inhibitors. On the other hand, they showed submicromolar hCA XII inhibition, with K_I_s in the range of 0.30–0.93 µM, making them highly CA XII-selective inhibitors.

## 1. Introduction

In most living organisms, the equilibrium between metabolically generated CO_2_ and bicarbonate is slow and needs a catalyst for supporting the metabolic requirements. This catalyst is the metalloenzyme carbonic anhydrase (CA, EC 4.2.1.1)^1–5^, of which a multitude of genetically distinct families have been described so far^6–9^. The CO_2_ hydration reaction also leads to the formation of a weak base (bicarbonate) and a strong acid (H^+^ ions) from two neutral molecules, being thus highly useful for pH regulation and several metabolic pathways^10–16^. Furthermore, CAs are among the most efficient catalysts known in nature, being able to catalyse the hydration of >10^6^ molecules of CO_2_ per second^1–5^. In vertebrates, including humans, only α-class CAs are present, with a high number of isoforms possessing a diverse subcellular/tissue localisation, catalytic activity and presumably physiologic roles were described so far^1–3^^,^^8^^,^^11^. The 15 human (h) CA isoforms are in fact involved in a multitude of diseases, and mainly their inhibitors have pharmacologic applications for the treatment of a range of diseases including glaucoma and other ophthalmologic problems, oedema, epilepsy, obesity, tumours, arthritis, etc^17–25^. Only sulphonamides and sulphamate CA inhibitors (CAIs) are in clinical use at this moment^1–3^^,^^5^^,^^23–25^, although many other different chemotypes were discovered in the last period to exert such an action, among which coumarins and sulphocoumarins^26–31^, phenols^20^^,^^22^, mono-/dithiocarbamates^32^, and carboxylates^33–38^. What is notable for these new chemotypes is the fact that they possess rather different inhibition mechanisms from the sulphonamides, which coordinate in deprotonated form to the metal ion from the CA active site^1–5^. On the contrary, many carboxylates, the coumarins and the sulphocoumarins (which act as prodrug CAIs^26–29^), inhibit CAs by diverse mechanisms^5^: they either anchor to the zinc-coordinated water molecule/hydroxide ion^35–37^, occlude the active site entrance^26^, or bind out of the active site^38^. Thus, this chemotype started to be quite investigated in the last period also because such derivatives are much more isoform-selective compared to the classical sulphonamide/sulphamate inhibitors^1–5^. This is mainly due to the fact that the binding sites not directly associated with the metal ion are less conserved among the many hCA isoforms, and as such non-classical inhibitors bind towards the exit of the active site, they interact with the non-conserved regions of the various isoforms, showing in this way a more selective inhibition profile compared to the sulphonamides and their isosteres^3^^,^^5^^,^^8–10^. Considering our interest in developing novel classes of isoform-selective CAIs, we report here a new class of carboxylates which show a selective inhibition profile of the tumor-associated isoform CA XII.

## 2. Experimental

### 2.1. Materials and methods

All starting materials, chemicals, reagents, and solvents were purchased from commercially known reputable sources and were used without further purifications. IR spectra (KBr, cm^−1^) were recorded on Shimadzu 8201 PC FTIR spectrophotometer (Shimadzu Ltd., Japan). ^1^H and ^13^C-NMR spectrum were recorded using (400 MH_Z_) JEOL-NMR spectrometer (JEOL Ltd., Tokyo, Japan), and the chemical shifts are reported in δ ppm. Elemental analyses were performed on PerkinElmer 2400 elemental analyser (PerkinElmer Inc., 940 Winter Street, Waltham, MA, USA), and the values found were within ±0.3% of the theoretical values. TLC silica (Type 60 GF254, Merck) and was visualised by UV light at 254 nm were used to check the purity of the compounds and monitoring the progress of the reaction. Melting points were recorded in open capillary tubes and are uncorrected (Sigma-Aldrich Chemie GmbH, 82024 Taufkirchen, Germany).

### 2.2. General procedure for the synthesis of 2,4-dioxothiazolidin acid derivatives 3a–g

The target products **3a–g** were prepared in three steps as follow:

- **Synthesis of thiazolidine-2,4-dione** (TZD) was prepared according to the reported method^39–41^: A mixture of chloroacetic acid (0.1 mol) and thiourea (0.1 mol) in water (10 ml) were placed in a 100 ml round bottom flask, the reaction mixture was stirred at rt for 30 min and then cooled down to 0 °C. To the reaction mixture, 8 ml of conc. HCl was added dropwise and after complete addition, the reaction mixture was refluxed for 16–18 h. The white solid was obtained after cooling, filtered and washed with water several times to remove the acid traces, dried and then the product TZD **1** was recrystallised from ethanol to afford white crystals mp 123–124 °C, in 91% yield (literature m.p. 123–125 °C)^39^.
- A solution of TZD **1** was treated with various appropriate aldehydes via refluxing in ethanol for 24 h in the presence of piperidine as a catalyst. The reaction mixture was poured into water followed by acidification with acetic acid to afford the products **2a–g**. The compounds **2a–g** were used directly to the next step without further purification for preparation of **3a–g**.
- A mixture of **2a–g** (1 mmol) and ethyl 2-bromoacetate (2 mmol) was refluxed for 24 h in acetone in presence of potassium carbonate (2 mmol) to furnish the target products obtained as white solid after evaporation of the solvent. The crude product was used directly to next step for preparation of the free carboxylic acid derivatives **3a–g** where the solid product was refluxed with glacial acetic acid and HCl in ratio (4:1) for 2 h to afford the pure (2,4-dioxothiazolidin-3-yl) acetic acid derivatives **3a–g** after evaporation of the solvent and then crystallised with ethanol. The spectral data for compounds **3a, 3f**, and **3g** were in agreement with the reported ones^42–45^.

#### 2.2.1. General procedure for the synthesis of 2,4-dioxothiazolidin-acetic acid derivatives

##### 2-(5-benzylidene-2,4-dioxothiazolidin-3-yl)acetic acid (3a)


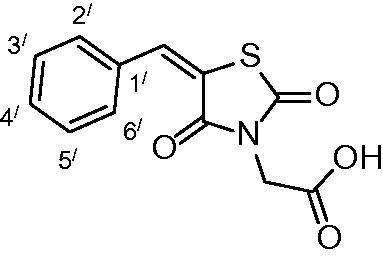


The product was obtained as a white crystal in 93% yield, mp: 214–216 °C. IR (KBr, cm^−1^): 3423 (OH of COOH), 2940 (CH-aliphatic), 1744, 1686, 1602 (CO). ^1^H-NMR (DMSO-d_6_, δ ppm): 4.40 (2H, s, CH_2_COOH), 7.51–8.00 (5H, m, Ar-H), 8.05 (1H, s, CH = C). ^13^C-NMR (DMSO-d_6_, δ ppm): 42.0 (*C*H_2_COOH), 114.3, 120.8, 130.0, 130.6, 133.7, 141.6, 163.1, 166.6, 167.4 (CO). Anal. Calc. for C_19_H_9_NO_4_S (263.27): C, 54.75; H, 3.45; N, 5.32. Found C, 54.96; H, 3.61; N, 5.53.

The product was obtained as light-yellow crystals in 96% yield, mp: 226–228 °C. IR (KBr, cm^−1^): 3371 (OH of COOH), 2950 (CH-aliphatic), 1733, 1685, 1600 (CO). ^1^H-NMR (DMSO-d_6_, δ ppm): 2.34 (3H, s, CH_)_ , 4.36 (2H, s, CH_2_COOH), 7.33 (2H, d, *J =* 6.6 Hz, Ar-H, H_3`_ & H_5`_), 7.51 (2H, d, *J =* 7.2 Hz, Ar-H, H_2`_ & H_6`_), 7.92 (1H, s, CH = C).^13^C-NMR (DMSO-d_6_, δ ppm): 21.6 (CH_3_), 42.7 (*C*H_2_-COOH), 113.9, 119.8, 130.5, 130.7, 134.4, 141.7, 165.5, 167.4, 168.4 (CO). Anal. Calc. for C_13_H_11_NO_4_S (277.3): C, 56.31; H, 4.00; N, 5.05. Found C, 56.44; H, 4.12; N, 5.26.

##### 2-(5-(4-chlorobenzylidene)-2,4-dioxothiazolidin-3-yl)acetic acid (3c)


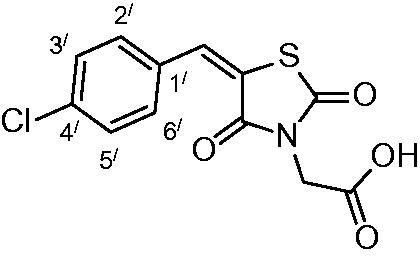


The product was obtained as light-yellow crystals in yield 92%, mp: 250–252 °C. IR (KBr, cm^−1^): 3383 (OH of COOH), 3008 (CH-aromatic), 1738, 1690, 1607 (CO). ^1^H-NMR (DMSO-d_6_, δ ppm): 4.42 (2H, s, CH_2_-COOH), 7.59–8.00 (4H, m, Ar-H), 8.03 (1H, s, CH = C).^13^C-NMR (DMSO-d_6_, δ ppm): 42.2 (*C*H_2_-COOH), 114.6, 121.3, 129.3, 131.5, 131.7, 132.4, 135.3, 142.1, 164.8, 166.5, 167.8 (CO). Anal. Calc. for C_12_H_8_ClNO_4_S (297.7): C, 48.40; H, 2.70; N, 4.70. Found C, 48.61; H, 2.83; N, 4.81

##### 2-(5-(2-chlorobenzylidene)-2,4-dioxothiazolidin-3-yl)acetic acid (3d)


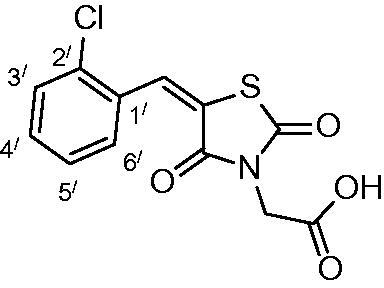


The product was obtained as white crystals in 90% yield, mp: 223–225 °C. IR (KBr, cm^−1^): 3364 (OH of COOH), 3064 (CH-aromatic), (2940) CH-aliphatic, 1490 (C = C), 1722, 1691, 1608 (CO). ^1^H-NMR (DMSO-d_6_, δ ppm): 4.43 (2H, s, CH_2_COOH), 7.55–7.69 (4H, m, Ar-H), 8.09 (1H, s, CH = C).^13^C-NMR (DMSO-d_6_, δ ppm): 42.0 (*C*H_2_COOH), 114, 124.1, 128.0, 130.8, 132.0, 137.4, 147.4, 164.3, 166.3, 167.6 (CO). Anal. Calc. for C_12_H_8_ClNO_4_S (297.7): C, 48.41; H, 2.71; N, 4.70. C, 48.65; H, 2.84; N, 4.95.

##### 2-(5-(4-bromobenzylidene)-2,4-dioxothiazolidin-3-yl)acetic acid (3e)


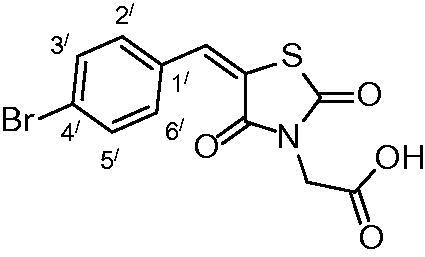


The product was obtained as yellowish white crystals in yield 94%, mp: 260–262 °C. IR (KBr, cm^−1^): 3371 (OH of COOH), 2948 (CH-aliphatic), 1696, 1606 (CO).^1^H-NMR (DMSO-d_6_, δ ppm): 4.37 (2H, s, CH_2_COOH), 7.56, (2H, d, *J* = 6.6 Hz, Ar-H, H_2`_& H_6`_), 7.73 (2H, d, *J* = 6 Hz, Ar-H, H_3`_ & H_5`_); 7.95(1H, s, CH = C). ^13^C-NMR (DMSO-d_6_, δ ppm): 42.8 (*C*H_2_-COOH), 114.2, 121.9, 124.9, 132.4, 132.8, 133.1, 142.4, 165.3, 167.1, 168.4 (CO). Anal. Calc. for C_12_H_8_BrNO_4_S (342.16): C, 42.12; H, 2.36; N, 4.09. Found C, 42.45; H, 2.59; N, 4.25.

##### 2-(5-(4-methoxybenzylidene)-2,4-dioxothiazolidin-3-yl) acetic acid (3f)


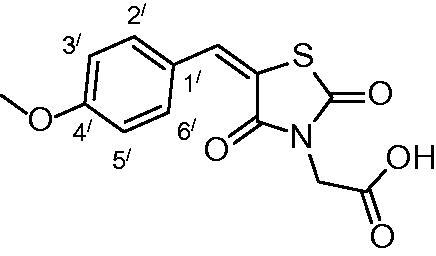


The product was obtained as yellowish white crystals in yield 91%, mp: 223–225 °C. IR (KBr, cm^−1^): 3368 (OH of COOH), 2926(CH-aliphatic), 1684, 1589 (CO). ^1^H-NMR (DMSO-d_6_, δ ppm): 3.81 (3H, s, OCH_3_), 4.35 (2H, 2, CH_2_COOH), 7.10 (2H, d, *J* = 7.2 Hz, Ar-H, H_3`_ & H_5`_), 7.60 (2H, d, *J=* 6.6 Hz, Ar-H, H_2`_& H_6`_), 7.92 (1H, s, CH = C). ^13^C-NMR (DMSO-d_6_, δ ppm): 42.7 (*C*H_2_COOH), 56.0 (OCH_3_), 115.5, 117.8, 125.7, 132.9, 134.3, 142.7, 161.8, 165.6, 167.4, 168.5(CO). Anal. Calc. for C_13_H_11_NO_5_S (293.29): C, 53.24; H, 3.78; N, 4.78. Found C, 53.09; H, 3.85; N, 4.93.

##### 2-(5-(3-methoxybenzylidene)-2,4-dioxothiazolidin-3-yl) acetic acid (3g)


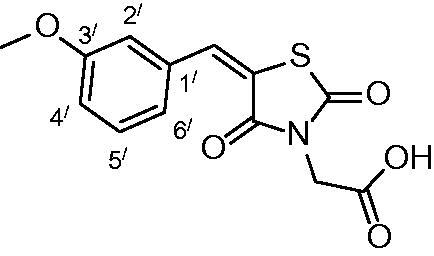


The product was obtained as light-yellow crystals in yield 93%, mp: 184–186 °C.

IR (KBr, cm^−1^): 3372 (OH of COOH), 2940 (CH-aliphatic), 1734, 1691, 1609 (CO). ^1^H-NMR (DMSO-d_6_, δ ppm): 3.78 (3H, s, OCH_3_), 4.36 (2H, s, CH_2_-COOH), 7.06 (1H, d, *J =* 7.8 Hz, Ar-H, H_4`_), 7.17 (2H, s, Ar-H, H_2`_ & H_6`_), 7.42 (1H, dd, *J =* 7.8 & 4.2 Hz, Ar-H, H_5`_), 7.93(1H, s, CH=C). ^13^C-NMR (DMSO-d_6_, δ ppm): 42.7 (*C*H_2_-COOH), 55.7 (OCH_3_), 116.0, 117.2, 121.5, 122.4, 130.9, 134.3, 134.5.142.7, 160.1, 165.4, 167.3, 168.4 (CO). Anal. Calc. for C_13_H_11_NO_5_S (293.29): C, 53.24; H, 3.78; N, 4.78. Found C, 53.09; H, 3.85; N, 4.93.

### 2.3. CA inhibition assay

An Applied Photophysics stopped-flow instrument has been used for assaying the CA catalysed CO_2_ hydration activity^46^. Phenol red (at a concentration of 0.2 mM) was used as indicator, working at the absorbance maximum of 557 nm, with 20 mM Hepes (pH 7.5) as buffer and 20 mM Na_2_SO_4_ (for maintaining constant the ionic strength), following the initial rates of the CA-catalysed CO_2_ hydration reaction for a period of 10–100 s. The CO_2_ concentrations ranged from 1.7 to 17 mM for the determination of the kinetic parameters and inhibition constants. For each inhibitor, at least six traces of the initial 5–10% of the reaction have been used for determining the initial velocity. The uncatalysed rates were determined in the same manner and subtracted from the total observed rates. Stock solutions of inhibitor (0.1 mM) were prepared in distilled–deionised water, and dilutions up to 0.01 nM were done thereafter with the assay buffer. Inhibitor and enzyme solutions were preincubated together for 15 min at room temperature prior to assay in order to allow for the formation of the E–I complex. The inhibition constants were obtained by nonlinear least-squares methods using PRISM 3 and the Cheng–Prusoff equation, as reported earlier^47–50^, and represent the mean from at least three different determinations. All CA isoforms were recombinant ones obtained in-house as reported earlier^51–53^.

## 3. Results and discussion

### 3.1. Chemistry

**Scheme 1.**
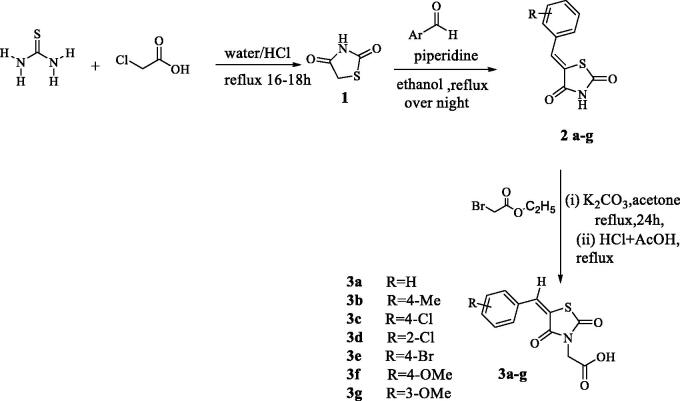
Preparation of 2,4-dioxothiazolidinyl acetic acids **3a–3g**.

Condensation of thiourea with chloroacetic acid afforded 2,4-dioxothiazolidine **1**, which was reacted with aromatic aldehydes leading to the alkenyl key intermediates **2**. These were further N-alkylated with methyl bromoacetate followed by removal of the methyl ester protection, which afforded the 2,4-dioxothiazolidinyl acetic acids **3a–g** (Scheme 1).

The nature of groups R attached to the aromatic ring was chosen in such a way as to induce chemical diversity, with both electron-attracting and electron-donating moieties being included in the new derivatives **3a–3g** generated by the above-described approach.

### 3.2. Carbonic anhydrase inhibition

Carboxylic acid derivatives **3** reported here were assayed for the *in vitro* inhibition of four major human CA isoforms, the cytosolic hCA I and II (widespread isoforms in a multitude of tissues and organs)^1–5^, and the tumor-associated, transmembrane ones hCA IX and XII, recently validated antitumor/antimetastatic targets^6^^,^^7^ (Table 1).

**Table 1.** CA inhibitory activity of carboxylates **3a–3g** and standard sulphonamide inhibitor acetazolamide AAZ, by a stopped-flow CO_2_ hydrase assay^45^.

K_I_ (µM)*				
	hCA I	hCA II	hCA IX	hCA XII
**3a**	>100	>100	22.2	0.58
**3b**	>100	>100	>100	0.93
**3c**	>100	>100	3.1	0.66
**3d**	>100	>100	24.1	0.47
**3e**	>100	>100	33.3	0.91
**3f**	>100	>100	3.2	0.85
**3g**	>100	>100	>100	0.30
AAZ	0.250	0.012	0.026	0.0057

* Mean from three different assays, by a stopped-flow technique (errors were in the range of ±5–10% of the reported values).

As shown from data of Table 1, unlike the standard sulphonamide acetazolamide, which is an efficient, nanomolar hCA I and II inhibitor, the carboxylic acids **3** did not inhibit these two isoforms (K_I_s > 100 µM), a situation also seen with other carboxylates such as the 2-hydroxy-cinnamic acids formed by the CAs catalysed hydrolysis of coumarins^26^. hCA IX was on the other hand inhibited in the high micromolar range by most of these derivatives, except **3b** and **3g** which had K_I_s > 100 µM. The best hCA IX inhibitors were **3c** and **3f** which have K_I_s of 3.1–3.2 µM and incorporate 4-chloro and 4-methoxy moieties in the aromatic part of the molecule. Structurally related derivatives such as **3a**, **3d**, and **3e** had inhibition constants in the range of 22.2–33.3 µM, being thus an order of magnitude less effective compared to **3c** and **3f**. Thus, very minor structural changes lead from a low micromolar to a high micromolar and to an ineffective hCA IX inhibitor (Table 1).

Surprisingly, hCA XII was effectively inhibited by all carboxylates 3, in the submicromolar range, with K_I_s of 030–0.93 µM. The structure-activity relationship is quite flat, since the difference in activity between these compounds is quite low. What is really remarkable is the fact that some of these CAIS are highly CA XII-selective, such as for example **3b** and **3g**, which do not significantly inhibit hCA I, II and IX, but are submicromolar inhibitors of CA XII, a profile not seen with other classes of compounds until now.

## 4. Conclusions

A small series of 2,4-dioxothiazolidinyl acetic acids was prepared from thiourea, chloroacetic acid, aromatic aldehydes and ethyl-2-bromoacetate. They were assayed for the inhibition of four physiologically relevant CA isoforms, the cytosolic hCA I and II, and the transmembrane hCA IX and XII, involved among others in tumorigenesis (hCA IX and XII) and glaucoma (hCA II and XII). The two cytosolic isoforms were not inhibited by these carboxylates, which were also rather ineffective as hCA IX inhibitors. On the other hand, they showed submicromolar hCA XII inhibition, with K_I_s in the range of 0.30–0.93 µM, making them highly CA XII-selective inhibitors.
